# A LabVIEW Platform for Preclinical Imaging Using Digital Subtraction Angiography and Micro-CT

**DOI:** 10.1155/2013/581617

**Published:** 2013-02-28

**Authors:** Cristian T. Badea, Laurence W. Hedlund, G. Allan Johnson

**Affiliations:** Department of Radiology, Center for In Vivo Microscopy, Duke University Medical Center, Durham, NC 27710, USA

## Abstract

CT and digital subtraction angiography (DSA) are ubiquitous in the clinic. Their preclinical equivalents are valuable imaging methods for studying disease models and treatment. We have developed a dual source/detector X-ray imaging system that we have used for both micro-CT and DSA studies in rodents. The control of such a complex imaging system requires substantial software development for which we use the graphical language LabVIEW (National Instruments, Austin, TX, USA). This paper focuses on a LabVIEW platform that we have developed to enable anatomical and functional imaging with micro-CT and DSA. Our LabVIEW applications integrate and control all the elements of our system including a dual source/detector X-ray system, a mechanical ventilator, a physiological monitor, and a power microinjector for the vascular delivery of X-ray contrast agents. Various applications allow cardiac- and respiratory-gated acquisitions for both DSA and micro-CT studies. Our results illustrate the application of DSA for cardiopulmonary studies and vascular imaging of the liver and coronary arteries. We also show how DSA can be used for functional imaging of the kidney. Finally, the power of 4D micro-CT imaging using both prospective and retrospective gating is shown for cardiac imaging.

## 1. Introduction

Clinical X-ray-based imaging using digital subtraction angiography (DSA) or computed tomography (CT) has highlighted the value of dynamic real-time acquisition for characterizing cardiac function and blood flow. Translating this imaging technology to preclinical studies has enormous potential to help study critical pathways in genetic models and to highlight potential concerns in drug safety evaluation. Therefore, one of the leading edges of X-ray-based preclinical imaging is the extension to faster scanning to allow collection of functional information such as in cine cardiac and perfusion studies. But small animal imaging poses formidable challenges that require both high spatial and temporal resolution. For example, the mouse heart has a diameter of about 5 mm and has heart rates as high as 600 beats/minute. The lack of commercial systems suitable for such tasks prompted us to develop a dual source/detector X-ray imaging system to use for both micro-CT and DSA studies in rodents [1]. The control of such a complex imaging system requires substantial software development for which we used the graphical language LabVIEW (National Instruments, Austin, TX). A key benefit of LabVIEW over other development environments is the extensive support for accessing instrumentation hardware. Drivers for many different types of instruments and buses are included or are available for use. A literature review shows multiple examples of LabVIEW implementations for different medical imaging tasks: CT reconstruction [2], real-time measurements of blood vessel motion [3], or simulations for medical imaging systems [4]. Others have reported using LabVIEW software for control and data acquisition in a cone beam micro-CT system [5], a phase contrast CT system [6], or even for a small animal conformal irradiator [7]. In this paper, we describe our approach to control custom-built, flexible X-ray-based imaging systems using applications developed in LabVIEW. 

## 2. Material and Methods

Successful in vivo small animal imaging requires a thoughtful integration of engineering, physiology, and end point application. The main components of our system include the dual source/detector X-ray imaging system, a mechanical ventilator, a physiological monitor, and a power microinjector for vascular delivery of X-ray contrast agents. All these components are controlled by LabVIEW applications that also support flexible sampling protocols as event sequencers that synchronize the details of image acquisition with contrast injections and cardiac and breathing activity. Here, we present the various components of our system and how they are controlled by LabVIEW applications. These applications were developed and run on a Dell PC with Intel Core 2 Quad Q9650 3 GHz processor and 3 GB RAM memory, with three boards (National Instruments)—two counter/timers PCI-6602 (8-channel counter/timer with digital I/O) and an NI-DAQ card PCI-6025E (multifunction data acquisition device with 16 analog inputs, 2 analog outputs). The PCI-6025E DAQ is used by the physiological monitor application, while the other two counter/timer boards are used to control the ventilator, the power microinjector, and all event sequencers for DSA and micro-CT.

### 2.1. Dual Source/Detector X-Ray System

For both DSA and micro-CT, imaging data are acquired with our dual source/detector X-ray system described in detail elsewhere [1]. The imaging system uses two Varian A197 X-ray tubes (Varian Medical Systems, Palo Alto, CA, USA) with dual focal spots of 0.3/0.8 mm. The tubes are commonly used in clinical angiography, as they provide high instantaneous flux and large heat capacity. Two Epsilon High Frequency X-ray generators (EMD technologies Quebec, Canada) are used to control both X-ray tubes. The system has two X-ray detectors with a Gd2O2S phosphor (XDI-VHR 2 115 mm, Photonic Science, East Sussex, UK) with 12 bit digitisation (16 bit extended dynamic range), pixel size of 22 microns, 115-mm input taper size, and 4008 × 2672 image matrix. Both detectors allow on-chip binning of up to 8 × 8 pixels and subarea readout to enable higher-speed readout of 10 frames/second, that is, a temporal resolution of 100 ms. The high-performance X-ray tubes/generators provide short (10 ms) exposures that minimize motion blurring with sufficient flux to fill the 12 bits of the detector digitizer. The X-ray tubes and detectors remain stationary in the horizontal plane. The animal is positioned vertically in a cradle and is incrementally rotated. The source to subject distance (702 mm) and subject to detector distance (100 mm) result in a geometric blur of the focal spot that matches the Nyquist sample at the detector [8]. A separate PC is dedicated to each X-ray detector providing high-speed image capture in 8 GB of on-board memory that serves as a fast frame storage. At the conclusion of each imaging sequence, data are downloaded to another computer where MATLAB routines (MathWorks, Natick, MA) perform the requisite after processing such as the subtraction for DSA or tomographic image reconstruction for micro-CT.

We have implemented LabVIEW applications that can control the critical X-ray exposure parameters (kVp, mA and ms) for our Epsilon High Frequency X-ray generators via the Serial Protocol RS-232. To achieve this control, we have used the Epsilon Development Kit that allows access to the serial protocol. The software control of the kVp has enabled us to implement kVp switching strategies for single source, dual energy micro-CT scanning [9].

### 2.2. Small Animal Physiologic Monitoring and Support

**Figure 1:**
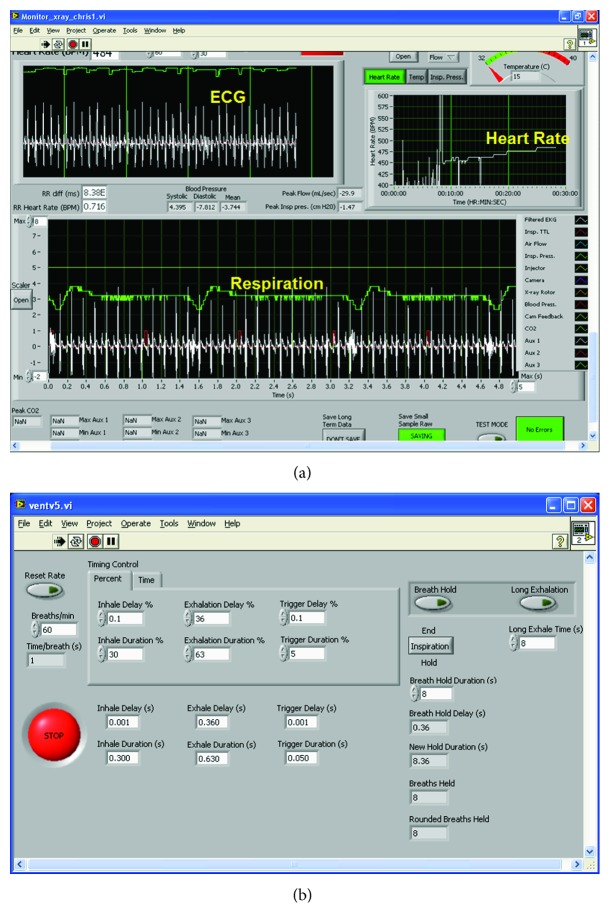
(a) Graphical user interfaces for physiologic monitoring and (b) ventilation controller using LabView.

Because the small animals must be chemically restrained during imaging, their physiologic status must be carefully monitored to ensure survival [10]. In our protocol, during imaging, rodents are anesthetized with isoflurane (1.5%) mixed with 50% oxygen and balanced with nitrogen, delivered via nose cone or by mechanical ventilation (see below). ECG is monitored with electrodes (Blue Sensor, Medicotest, UK) taped to the foot pads, and body temperature is maintained with heat lamps, a rectal thermistor, and feedback controller (Digi-Sense, Cole Parmer, Chicago, IL). A pneumatic pillow under the thorax is used to monitor respiration. We use Coulbourn modules to acquire signals to monitor cardiac (ECG) and breathing (pneumatic pillow) activity and these signals are processed via our LabVIEW monitor application and displayed on a computer monitor (see Figure 1(a)). The Coulbourn modules include a comparator that generates transistor-transistor logic (TTL) signals based on the R peaks in the ECG and peaks of the breathing waveforms. 

The monitor application also generates TTL pulses used to trigger image acquisition for cardio-respiratory gating. The triggers are adjusted to occur at specific times during the cardiac and breathing cycles for gating. For example, trigger pulses can be adjusted to occur at peak inspiration or at end expiration for lung imaging or at the R peak of ECG for cardiac imaging. 

### 2.3. Mechanical Ventilators

In cardiopulmonary imaging studies, small animals may require mechanical ventilation and endotracheal intubation to not only support gas exchange for lung injury models but also to control breathing motion blurring and misregistration of cardiopulmonary and upper abdomen anatomy [11]. Mechanical ventilation is also useful for delivery of inhalant anesthesia such as isoflurane especially for survival studies [10]. We have constructed a small animal ventilator specifically for these uses [12]. A pneumatically-controlled inspiration/expiration valve is attached directly to an endotracheal tube (thus minimizing dead volume to a few microliters), and breathing gas supply and pneumatic control hoses can be of extended length facilitating connection to the animal in the cradle. This arrangement allows positioning the electropneumatic control valves, flow regulators, and vaporizer several feet from the animal. Our ventilator LabVIEW application controls all the important ventilation parameters such as breathing rate, duration of inspiration/expiration, and time of TTL output pulses for synchronizing imaging acquisition to any phase of the breathing cycle (see Figure 1(b) of front panel). 

### 2.4. Microinjector

DSA is an important preclinical imaging tool for studying vascular diseases involving clots, stenoses, and hemorrhage to name a few. Implementing DSA and contrast-enhanced micro-CT imaging procedures for small animals using conventional clinical contrast agents is a daunting task that requires coordination of image acquisition with injection of a radiopaque contrast media in microliters amounts for a few milliseconds and synchronized to phases of the cardiac or respiratory cycles. This can only be achieved, as it is clinically, with high power injectors and precise computer control. Such instrumentation did not exist for small animals until we custom-built such a system which is described in detail in [13]. The system consists of a contrast microinjector powered by compressed nitrogen and controlled by a solenoid valve capable of high flow rates and millisecond response time. The contrast agent is preheated to reduce viscosity and thermal shock. The microinjector is controlled via our LabVIEW DSA sequencer (described in the next section). Note that in many of our studies, to provide the necessary blood/tissue contrast, we also use a liposomal blood pool contrast agent [14] containing 123 mg I/mL delivered by hand injection via a tail vein catheter at a dose of 0.012 mL/g body weight.

#### 2.4.1. DSA Event Sequencer

**Figure 2:**
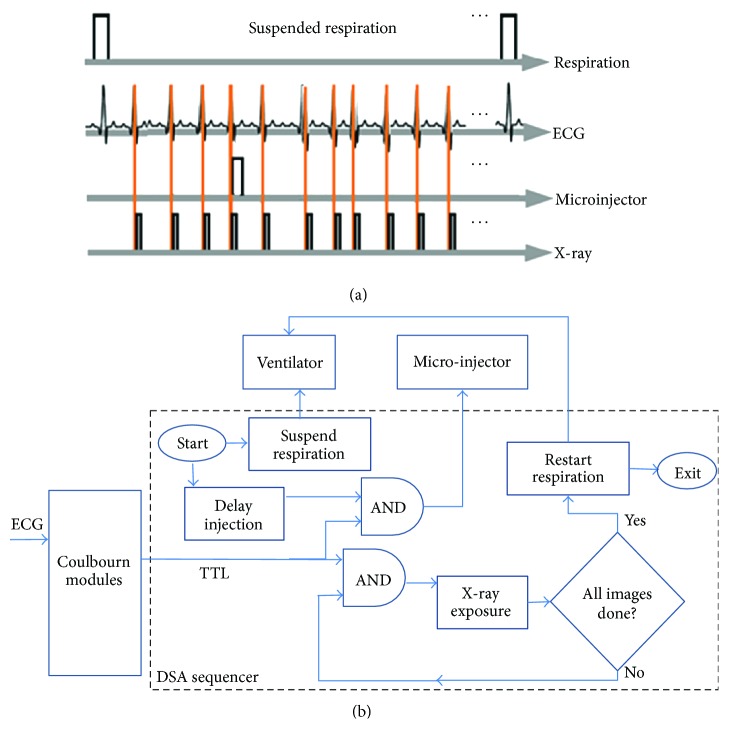
DSA scanning. In (a) we present the sampling diagram and in (b) we show the flowchart of the DSA event sequencer application developed in LabVIEW. DSA images are acquired before and after contrast injection synchronized to ECG.

DSA allows functional imaging using iodinated contrast agent Isovue-370 (Bracco Diagnostics, Princeton, NJ). In rodents, DSA X-ray projection images are acquired every successive heartbeat at about 8–10 frames/sec with spatial resolution of 88–100 microns.  Figure 2 shows the sampling diagram (a) and the flowchart of the DSA event sequencer.

Ventilation, X-ray exposure, and contrast injection are all precisely synchronized using the DSA event sequencer. The sequencer uses the outputs from the Coulbourn module that conditions and thresholds the ECG signal resulting in TTL pulses, to control both the image acquisition and the micro-injection. The injection and X-ray exposures are thus synchronized to the R wave of the ECG. A DSA imaging sequence for cardiopulmonary imaging is started by stopping the ventilator during end expiration for a few seconds (typically between 5 and 10 seconds) via control of the ventilator application. Suspended respiration minimizes respiratory artifacts in the subtracted DSA images. A set of noncontrast images is acquired, averaged, and used to create a mask. The injection is then initiated by a command to the microinjector which is followed immediately by the acquisition of postcontrast injection images. At the end of the DSA run, the ventilator is restarted. After sampling, a subtraction of the post-contrast projections and the pre-contrast mask provide exquisite DSA images of vasculature in the small animal.

We have used DSA to assess cardiopulmonary blood flow [15] and effects of the vasoconstrictor, phenylephrine [16]. In these studies, contrast was injected via a catheter placed in the jugular vein. In DSA imaging of the liver, we have used two catheters. The first catheter was used to visualize the portal vein system and was inserted into a mesenteric vein through laparotomy and advanced into the portal vein. To view the arterial system, a second catheter was placed into the left common carotid and into the abdominal aorta to just above the celiac artery. 

For DSA imaging of the coronary arteries in a rat heart, a catheter was inserted into the right common carotid artery and advanced so the tip was just cranial to the aortic valve using real-time blood pressure guidance. Contrast injection was timed, using ECG, to occur when the aortic valve was closed; thus, contrast agent flowed into the coronary arteries as it normally occurs. We have used this procedure to measure changes in coronary artery dilation and myocardial perfusion induced by a vasodilator drug, nitroprusside [17]. 

DSA can be used not only for morphological vascular imaging but also as a functional tool to assess organ or tumor perfusion. We have applied DSA to study, for example, cardiopulmonary blood flow and kidney perfusion in rats. Renal permeability via Patlak analysis was used as a method to assess renal function with images provided by dynamic CT [18]. Permeability within the kidney is largely determined by the filtration at the glomerulus level. A rat was endotracheally intubated and mechanically ventilated. A catheter was placed in the abdominal aorta through the left carotid artery with the tip at the level of celiac artery and contrast agent was injected using the power microinjector. We injected 1 mL of Isovue 370 mg/mL I contrast agent over 1 second. DSA images were acquired every heartbeat (temporal resolution around 140–150 ms) and a spatial resolution of 100 microns. A total of 40 images were acquired in a DSA run from which the first 6 were acquired before injection and were used to create the mask image that is subtracted from the postcontrast images.

We have used ROI Manager available in ImageJ (http://rsbweb.nih.gov/ij/) to measure DSA enhancement in regions of interests (ROIs) selected in the kidney's cortex and medulla as well as in renal artery and the aorta. Perfusion can be estimated as the peak enhancement over area under the curve for aorta [19]. Permeability was obtained with the Patlak analysis [20]. A Patlak plot consists in plotting *C*(*t*)/*Cb*(*t*) on *y*-axis and ∫(*Cb*(*t*))/*Cb*(*t*) on *x*-axis, where *Cb*(*t*) is the blood concentration at time *t*, here taken in the descending aorta, and *C*(*t*) is the concentration of contrast medium in the kidney's cortex or medulla at time *t*. The intercept of the regression lines in the Patlak plots represent rbv (residual blood volume, i.e., fraction of the volume that is blood) while the slope represents permeability information.

#### 2.4.2. Micro-CT Event Sequencers

**Figure 3:**
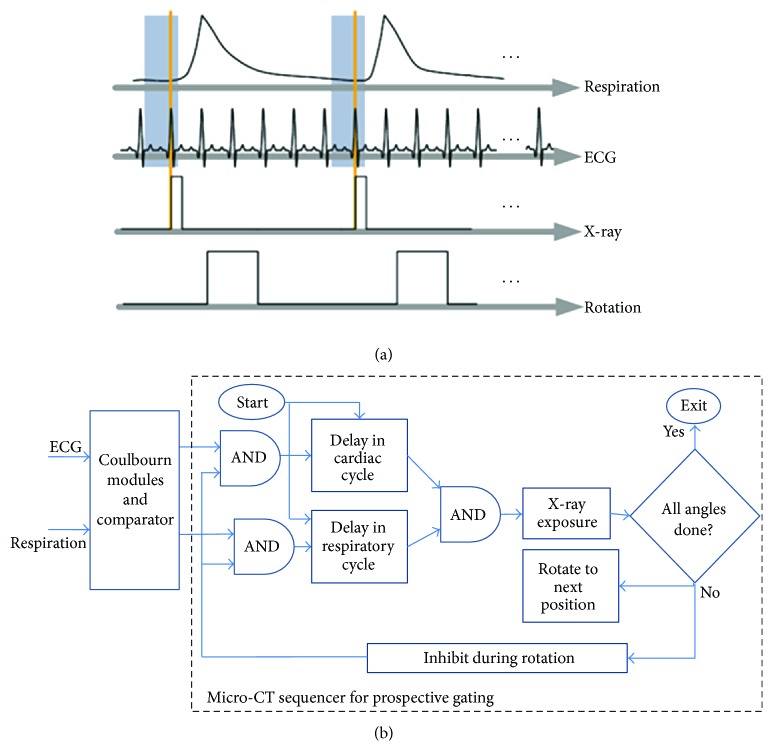
Micro-CT with prospective cardiorespiratory gating. (a) presents the sampling diagram, and (b) shows the flowchart of the Sequencer application developed in LabVIEW. The gray zones in (a) are the windows in end expiration in which the R peaks in the QRS cycle are triggering the acquisition.

Cardiorespiratory gating plays an essential role in minimizing motion blurring and misregistration during in vivo micro-CT scanning. We have developed a number of LabVIEW applications that implement either prospective or retrospective gated acquisitions. Micro-CT requires incremental rotation (e.g., 1° angular step) of the animal achieved using an Oriel Model 13049 digital stepping motor.  Figure 3 presents the sampling diagram and the flowchart for the micro-CT sequencer implementing the prospective cardio-respiratory gating [21]. In this protocol, the X-ray exposures are triggered by the coincidence of a selected respiratory phase (end inspiration or end expiration) and a selected cardiac phase, for example, R peak in the ECG cycle.  Figure 3(b) shows a simplified flowchart of the LabVIEW implementation. The program receives the respiratory and ECG signals from the Coulbourn modules. These signals are conditioned, and a threshold is applied to select the R peaks and respiratory peaks and to deliver TTL pulses. Using counters/timers, we then apply delays to select the respiratory and the cardiac phase of interest and use them as inputs to an AND operation. Their temporal coincidence results in a TTL pulse that triggers the X-ray exposure followed by a rotation to the next angle during which the ECG and respiration peaks are inhibited. This acquisition protocol results in a set of projections with a constant angular steps of 1°. Consequently, the reconstructed micro-CT images obtained with filtered backprojection reconstruction algorithms such as Feldkamp [22] are generally of high quality and free of streaking artifacts. For cine-cardiac micro-CT studies, up to 10 cardiac phases in the cardiac cycle need to be acquired. With prospective gating, this is achieved sequentially by acquiring full sets of projections corresponding to each cardiac phase by changing the delays. The delays are updated in time as a percentage of RR intervals. However, because of the time spent waiting for the coincidence of cardiac and respiratory events, the scan time with prospective gating for a cine-cardiac study can take as long as 1 hour to cover 10 different phases of the cardiac cycle. 

**Figure 4:**
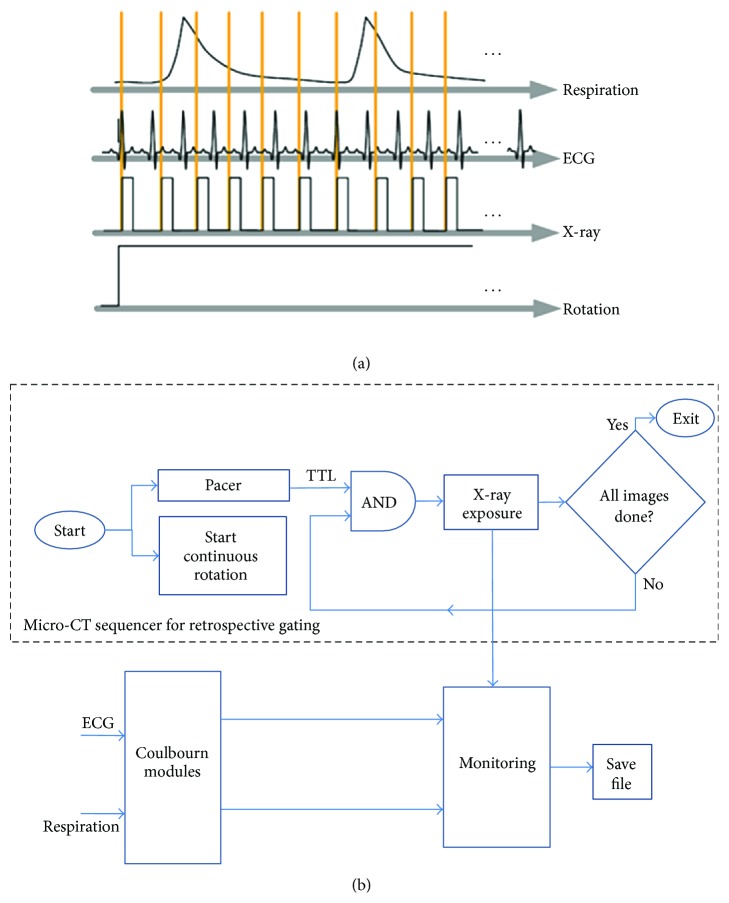
Micro-CT with retrospective gating. (a) presents the sampling diagram, and (b) shows the flowchart of the Sequencer application developed in LabVIEW.

Much faster sampling is achieved using retrospective gating. The sampling diagram and the flowchart of the LabVIEW sequencer are shown by Figure 4. In this sampling strategy, all input signals, including the ECG, respiration, and the X-ray exposure, are monitored in synchrony with acquisition and saved in a file. The acquisition is controlled by a pacer application, which generates TTL pulses (see Figure 4(b)). The frame rate for camera readout limits us to a maximum of 10 frames/second. The acquisition starts by sending a command for a continuous rotation over 360°. When all projection images are acquired the script stops. To achieve sufficient number of projections for a cine-cardiac micro-CT, we perform 4 or 5 such rotations per study. The total sampling time is in between 5 to 8 minutes. Post-acquisition, the projection images are sorted and associated with angle, cardiac, and respiratory phase information using a MATLAB. Each cardiac cycle is divided into *N* intervals (each equal to 10% of the RR interval in our case for *N* = 10). Each projection is temporally registered with the ECG signal by finding the temporal distance from the previous R peak that was closest to the projection sampling time and dividing the distance by the RR period. This information is kept in files used during reconstruction. Retrospective gating results in an irregular angular distribution of projections that can create artifacts when filtered backprojection types of reconstruction algorithms are used. We have designed several reconstruction approaches to address this problem by using interpolations and bilateral filtering [23], point spread function deconvolution [24], or total variation CT [25]. 

**Figure 5:**
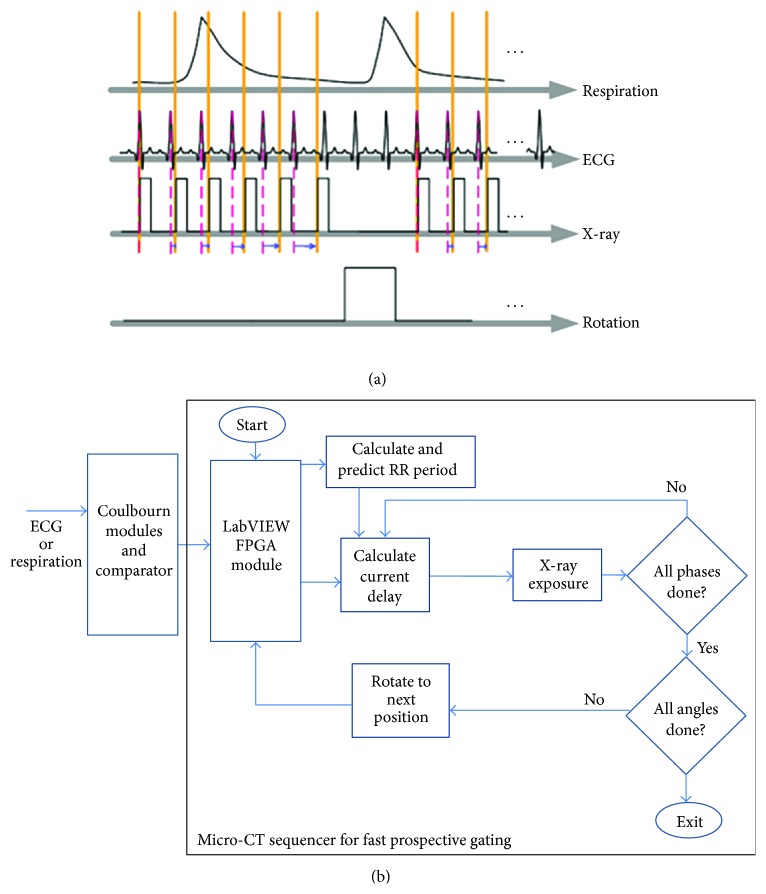
Micro-CT with fast prospective gating. (a) presents the sampling diagram, and (b) shows the flowchart of the Sequencer application developed in LabVIEW.

Recently, we have added a new gating strategy to our portfolio that combines advantages from prospective (regular angular distribution of projections) and retrospective gating (fast scanning). We call this strategy fast prospective gating (FPG) [26], and we show its sampling diagram and the flowchart of its LabVIEW implementation in Figures 5(a) and 5(b). Fast prospective gating involves computing variable delays on the fly, so that projections corresponding to multiple phases (cardiac or respiratory) are acquired in a rapid sequence at the same angle. When all the necessary projections are acquired, we rotate to the next angle and repeat the process. To meet real-time requirements of FPG for processing, we implemented the new acquisition protocol on a reconfigurable data acquisition card (PCI-7811R, National Instruments, TX). The card has R-series digital reconfigurable inputs/outputs at rates up to 40 MHz with a Virtex-II 1 M field-programmable gate array (FPGA) chip for onboard processing. The chip is integrated with LabVIEW and can be programmed using the LabVIEW FPGA module, which creates very high definition language (VHDL) code from the graphical code. The VHDL code is then transferred to the Xilinx tool chain to create a bit file, which is uploaded to the field-programmable gate array chip. Since multiple control loops and computations can be executed independently, we can achieve high loop rates and precise counting. The ECG or respiration signals coming out from our Coulburn module as a digital TTL pulses are directed to the digital I/O port of the PCI-7811R card. When a rising edge is detected, the time elapsed since the previous rising edge determines the RR interval. To predict the next RR period used to calculate the phase delay, we use an exponential moving average. This recursive method allows us to adapt the sampling rate over the course of the scan for animals with irregular heart rates. Using the chip's 40 MHz timer on the PCI-7811R board, the delay for each phase can be calculated by a single-cycle timed loop in 25 ns with a loop timer. When the time elapsed since the last R peak equals the delay, the field-programmable gate array generates a predefined output to trigger the exposure of the X-ray tube. Once the projection images corresponding to all of the desired cardiac phases are acquired, the sampling program sends a trigger signal to the rotation stage to rotate to the next angular position and start the acquisition process over again. This is repeated until the full 360° arc is complete. 

## 3. Results

**Figure 6:**
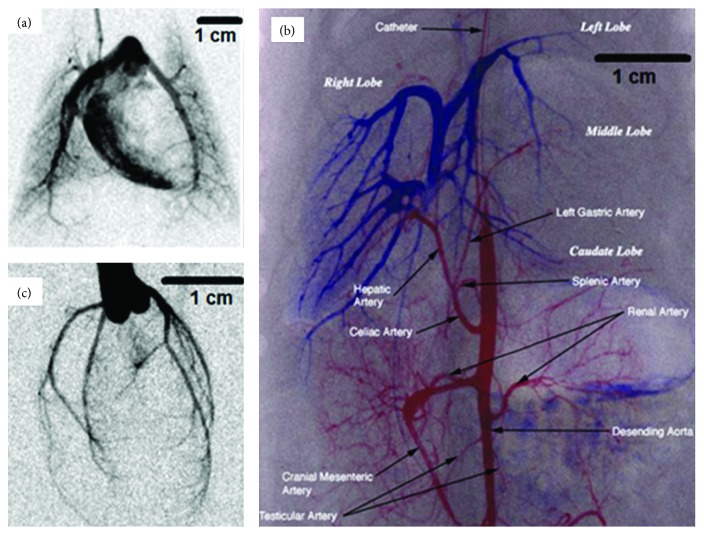
Examples of DSA imaging in a rat. (a) Pulmonary DSA obtained with a jugular vein injection with the catheter tip near the right atrium and contrast is seen in the right ventricle and major pulmonary arteries. (b) DSA of the liver and the lower abdomen. For imaging the portal vein system (blue), one catheter was inserted through a mesenteric vein and advanced into the portal vein. To view the arterial system (red), a second catheter was placed into the left common carotid and into the abdominal aorta to just above the celiac artery. Major vessels and sections of the liver are labeled. (c) Coronary artery DSA obtained with the catheter tip just cranial to the aortic valve and injecting contrast when the valve is closed. All major arteries are seen as well as some secondary and tertiary branches.

Our results present a series of images from DSA and micro-CT imaging using our integrated imaging system.  Figure 6(a) is an example of DSA of cardiopulmonary perfusion in a rat. The right ventricle and main pulmonary arteries are clearly seen.  Figure 6(b) illustrates the power of DSA-based vascular imaging in a rat liver using two catheters. The first catheter was used to visualize the portal vein system (blue) and the second catheter was used to view the arterial system (red), Major vessels and sections of the liver are labeled. Finally, Figure 6(c) presents a rat coronary artery DSA image. 

**Figure 7:**
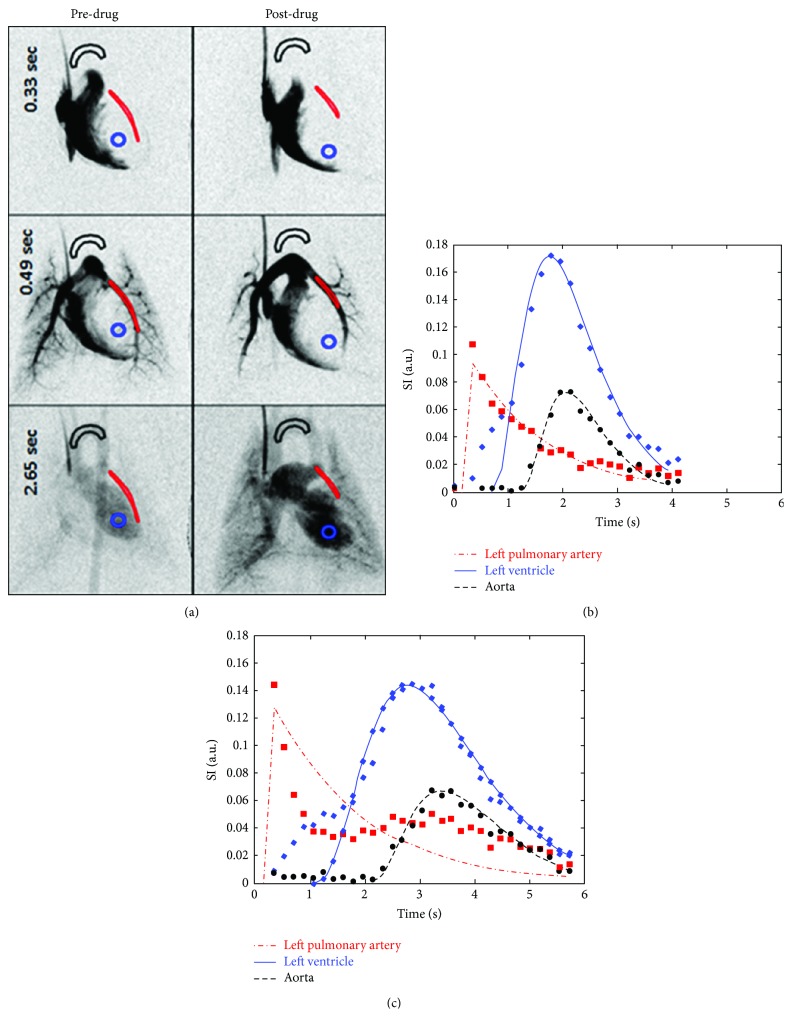
DSA imaging to test the effects of phenylephrine on a rat's cardiopulmonary system. The two columns in (a) show selected DSA images corresponding to pre-drug phase and a corresponding series 2 minutes after drug infusion. Three ROIs were selected in the aorta, left pulmonary artery, and left ventricle. The changes over time in mean attenuation values for these ROIs are shown for pre-drug (b) and post-drug (c) illustrating that drug-induced vasoconstriction delayed the flow of blood from right ventricle to aortic outflow in addition to distention of pulmonary arteries and left ventricle. We have fitted gamma variate curves to the measurement points in (b) and (c).

Figure 7 provides an example of DSA imaging applied for measuring real-time physiological changes before and after administration of the vasoconstrictor, phenylephrine, in a rat. Only 3 images at selected series from the complete DSA sets are shown in (Figure 7(a)). The progression of contrast can be followed from the right ventricle, through the lungs, and finally to the left ventricle and aortic arch. Phenylephrine infusion markedly alters both vessel anatomy and blood flow. The time attenuation curves for 3 ROIs in the aorta, left pulmonary artery, and left ventricle are shown in Figure 7(b) (for pre-drug) and Figure 7(c) (post-drug) show the progression of contrast through the cardiopulmonary system quantitatively. The plots ((b) versus (c)) clearly show that the vasoconstrictor effect of the drug is to delay the flow of blood from the right ventricle to the aorta and also distension of the pulmonary arteries suggesting pulmonary hypertension. 

**Figure 8:**
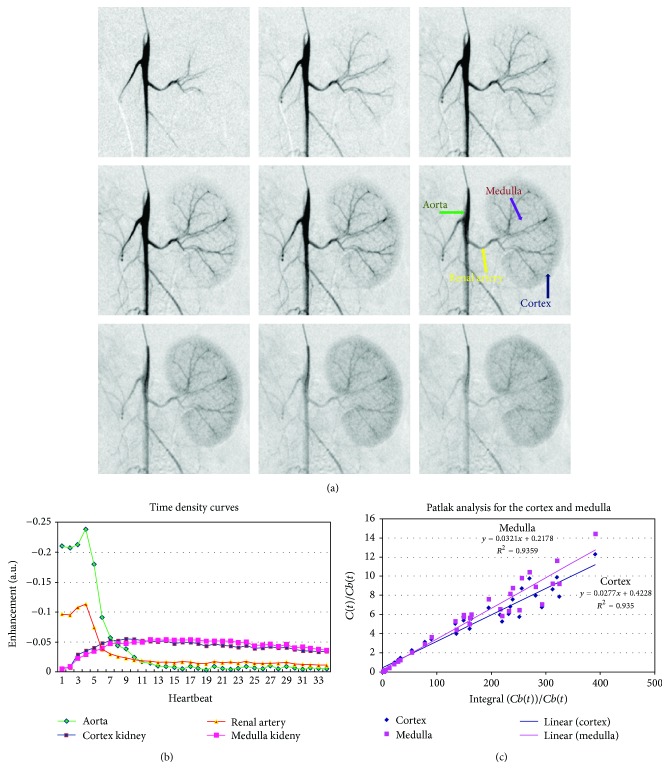
A DSA kidney perfusion study in a rat. (a) DSA images of the first 9 heartbeats after injection are shown. Note the arterial phase as well as the perfusion phase. (b) The time attenuation curves for selected ROIs in the cortex, medulla, renal artery, and aorta. (c) Patlak analysis for the cortex and medulla.

Figure 8 presents results from a kidney perfusion study in a rat.  Figure 8(a) shows first 9 images post-injection of the left kidneys. Note these images show the arterial phase (first images) as well as the perfusion phase and venous (last images). The selected ROIs are shown by Figure 8(a) and the time density (or attenuation) curves obtained by plotting the mean enhancements at each heartbeat are shown by Figure 8(b). Note that we had 34 heartbeats after injection. The Patlak plots are shown in Figure 8(c), together with their regression lines. The results for perfusion, rbv, and permeability of the cortex or medulla volume are listed in Table 1. Note slightly larger values for perfusion, as well as an increased rbv, in the cortex compared to medulla. But the opposite is true for permeability, that is, larger values are measured in medulla and this is consistent with CT-based analysis in humans [27]. 

**Figure 9:**
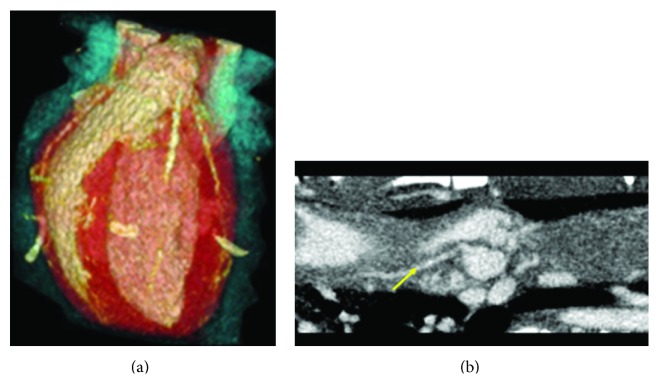
In vivo micro-CT with cardio-respiratory gating allows 3D visualization of the coronaries in a rat heart. Both (a) a 3D volumetric rendering and (b) curved reformation are shown. The arrow indicates a coronary artery.

Examples of cardiac micro-CT images in a rat using prospective cardio-respiratory gating are shown by Figure 9. Due to successful gating, the coronary arteries are visible both in the 3D volume rendering (Figure 9(a)) as well as in the curved reformation obtained in Osirix software (http://www.osirix-viewer.com).

**Figure 10:**
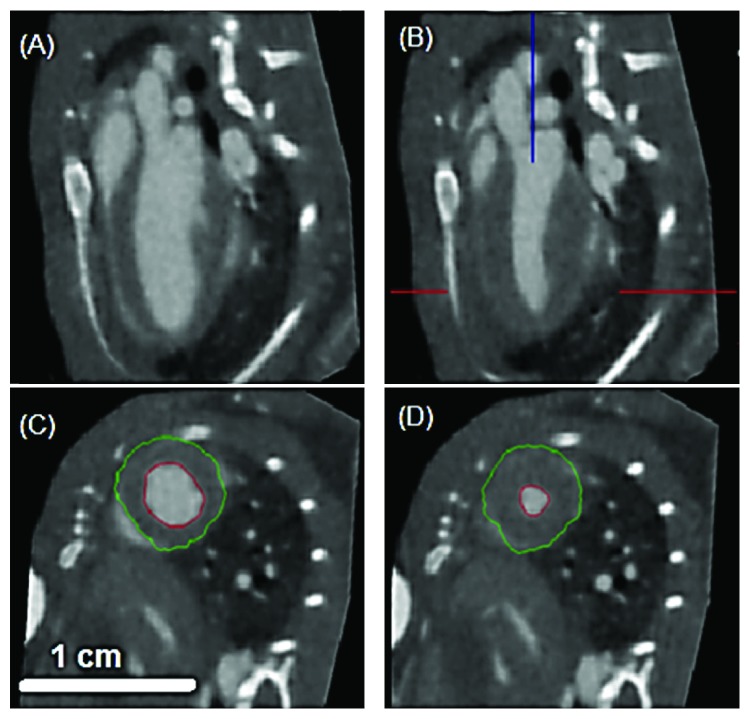
Examples of 4D murine cardiac micro-CT images using retrospective gating. The images show both slices cutting through the left ventricle in sagittal and oblique orientations in diastole ((A) and (C)) and systole ((B) and (D)).

In Figure 10, we present examples of mouse cardiac data acquired with retrospective gating. The figure shows both slices through the left ventricle in sagittal and oblique orientations in diastole ((A) and (C)) and systole ((B) and (D)). The 3D volumes in diastole and systole can be used to determine via segmentation the volumes of the left ventricle (see the delineated endocardium and epicardium) and use them to compute measures of cardiac function such as ejection fraction, stroke volume, and cardiac output [28].

## 4. Discussion and Conclusions

Small animal imaging has a critical role in phenotyping, drug discovery, and in providing a basic understanding of mechanisms of disease. Translating imaging methods from humans to small animals is not an easy task. In this paper, we describe our LabVIEW-based approach to building the software control of our custom built X-ray based preclinical imaging system. Both micro-CT and DSA modalities have been enabled by our system and used to provide both morphologic and functional data using dynamic acquisitions. DSA has shown its power in cardiopulmonary imaging and vascular imaging, in general. We have been able to visualize the coronary arteries in small animals—a very challenging task due to their small size, rapid motion of the heart, and the requirement that contrast injection occurs when the aortic valve is closed. To the best of our knowledge these challenges have not been overcome with commercial small animal X-ray or micro-CT systems that use microfocus X-ray tubes for which the exposure must be too long to achieve. The renal study described here is an intriguing example of the functional abilities of DSA that can be performed very rapidly. Additional work is required to validate the absolute measurements, but temporal changes induced pharmacologically are readily done now. Finally, our 4D micro-CT allows a wide range of gating protocols (prospective, retrospective, and fast prospective) providing particularly powerful opportunities for cardiac studies where sensitive measures of cardiac function are needed.

The LabVIEW programming environment, as demonstrated by our examples, enables creative solutions to otherwise vexing problems. The modular character of LabVIEW code enables us to reuse the code without modifications in many instances. Finally, the graphical nature of the language and simplicity of the graphical user interfaces have made LabVIEW the perfect choice for our DSA and micro-CT system as well as the peripheral tools such as the ventilator, the monitor, and power microinjector applications. This integrated imaging system has been in use now for more than 4 years and constitutes a major platform for current and future novel scientific explorations. 

## Figures and Tables

**Table 1:** The perfusion, rbv (residual blood volume), and permeability for the cortex and medulla in a rat kidney. Values are given in arbitrary units.

	Cortex	Medulla
Perfusion	0.0555	0.0544
rbv	0.4228	0.2178
Permeability	0.0277	0.0321
